# F246. A SYSTEMATIC REVIEW COMPARING THE NEURAL CORRELATES OF EMPATHY ASSOCIATED WITH THE ONSET AND PROGRESSION OF SCHIZOPHRENIA

**DOI:** 10.1093/schbul/sby017.777

**Published:** 2018-04-01

**Authors:** Meneshka Ponnampalam

**Affiliations:** The University of Birmingham

## Abstract

**Background:**

Empathic deficits present in nearly all Schizophrenia patients (SCZ). These result from impairments in various social cognitive tasks, often leading to social isolation and withdrawal. There is evidence that empathy deficits occur before illness-onset in those at ‘ultra-high risk’ of psychosis (UHR) and those with a ‘first-episode of psychosis’ (FEP). Empathy defects are associated with neurological abnormalities, which have been studied separately in UHR, FEP and SCZ populations. This review aims to gain further insight into neurological changes associated with illness progression, by comparing brain changes associated with empathy across UHR, FEP and SCZ populations.

**Methods:**

Studies considering functional activity, connectivity and structural changes in UHR, FEP and SCZ populations were systematically reviewed. Data from 26 studies was used.

**Results:**

All three subgroups showed abnormal patterns of activation and connectivity across a range of regions, particularly in the frontal, limbic and temporal areas. Structural abnormalities appeared as widespread grey matter loss, largely in the temporal lobe, across all three participant groups. Notably, impaired empathic behavioural responses were found in FEP and SCZ subjects only, despite abnormal brain patterns in all three groups.

**Discussion:**

Our findings suggest that abnormal connectivity, structure and activation of the frontal, limbic and temporal areas contribute significantly to empathy deficits, and also worsen with illness progression. However, the multifaceted nature of empathy means that behavioural impairments likely result from a combination of disruptions of the frontal, limbic and temporal areas as well as many other neural networks involved in social information processing.

